# Depletion-free Automated Enrichment of Serum Glycopeptides for High-Throughput Clinical Glycoproteomics

**DOI:** 10.1016/j.mcpro.2026.101611

**Published:** 2026-06-25

**Authors:** Jung Hoon Choi, Geul Bang, Jong Hyun Seol, Seong Woo Choi, Su-Min Lee, Bao Tan Nguyen, Seungjin Na, Cheol-Jung Lee, Heeyoun Hwang, Jinsoo Chung, Sang-Jae Park, Sang Myoung Woo, Hye Gwang Jeong, Jin Young Kim

**Affiliations:** 1 Center for Target-to-Therapeutics Research, Korea Basic Science Institute (KBSI), Ochang, Republic of Korea; 2 College of Pharmacy, Chungnam National University, Daejeon, Republic of Korea; 3 Department of Bio and Brain Engineering, Korea Advanced Institute of Science and Technology (KAIST), Daejeon, Republic of Korea; 4 Convergent Analytical Science, University of Science and Technology (UST), Daejeon, Republic of Korea; 5 Research Unit for Molecular Structure and AI, Korea Basic Science Institute (KBSI), Ochang, Republic of Korea; 6 Center for Urologic Cancer, National Cancer Center, Goyang, Republic of Korea; 7 Center for Liver and Pancreatobiliary Cancer, National Cancer Center, Goyang, Republic of Korea; 8 Critical Diseases Diagnostics Convergence Research Center, Korea Research Institute of Bioscience and Biotechnology (KRIBB), Daejeon, Republic of Korea; 9 College of Pharmacy, Chung-Ang University, Seoul, Republic of Korea

**Keywords:** automated glycopeptide enrichment, depletion-free, high-throughput glycoproteomics, N-glycoproteomics, serum, site-specific glycosylation

## Abstract

High-throughput and reproducible enrichment of N-glycopeptides remains a major bottleneck in large-scale and clinical glycoproteomics, despite substantial advances in mass spectrometry. Here, we present a depletion-free, high-throughput N-glycopeptide enrichment workflow (3kMAX) that combines 3 kDa molecular weight cut-off filtration with positive-pressure–driven mixed anion exchange (MAX) enrichment in a 96-well format. This platform enables parallel processing of up to 96 samples with minimal operator intervention, improving analytical throughput, robustness and inter-batch reproducibility. Systematic evaluation using human serum demonstrated that the combined workflow achieved superior glycopeptide selectivity and identification depth compared with conventional manual MAX enrichment or molecular weight cut-off filtration alone, without introducing detectable glycan-type bias. Sensitivity analysis across a fourfold range of serum input volumes (5–20 μl) showed stable identification performance and quantitative reproducibility, with 10 μl identified as an optimal standardized input. Day-to-day reproducibility assessments further confirmed consistent quantitative performance across different acquisition densities. Application of the workflow to hepatocellular carcinoma serum samples enabled reproducible, large-scale quantitative glycoproteomic profiling and revealed extensive disease-associated glycosylation remodeling, including site-specific regulation within individual proteins. Collectively, the 3kMAX platform provides a scalable solution for site-specific serum N-glycoproteomics and facilitates its translation into clinical and biomarker-oriented applications.

## Highlights

• Standardized workflow integrating MWCO pretreatment and automated MAX enrichment.
• 3kMAX enables high-throughput, reproducible processing of 96 serum samples.
• 3kMAX improves glycopeptide coverage without introducing glycan-type bias.
• Enables site-specific glycoproteomics from ≤10 μl serum in clinical cohorts.

## Graphical Abstract

**Figure:**
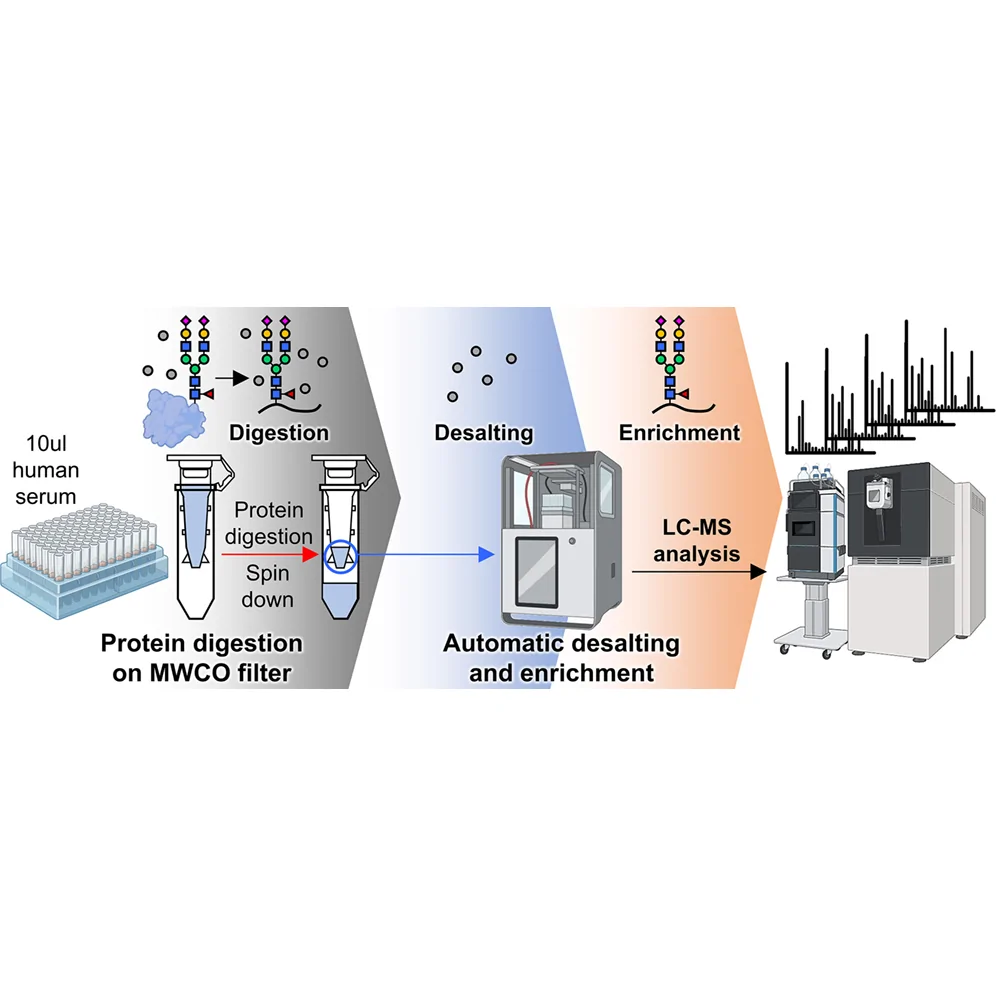

## In Brief

We developed 3kMAX, a depletion-free and automated and standardized workflow combining 3 kDa MWCO filtration and automated MAX enrichment in a 96-well format. The platform enables high-throughput, reproducible processing of serum samples with improved identification depth and minimal bias. Application to clinical samples demonstrated its utility for large-scale quantitative glycoproteomics and site-specific analysis revealing discrimination between normal and hepatocellular carcinoma (HCC) serum samples. This workflow establishes a practical and standardized foundation for clinical glycoproteomics and future biomarker discovery studies.

N-glycosylation is among the most abundant and functionally important post-translational modifications in eukaryotic cells, playing essential roles in protein folding, stability, trafficking, and cell–cell communication (1, 2, 3). Owing to their extensive structural diversity and microheterogeneity, N-glycans are highly sensitive to physiological and pathological perturbations, making them informative indicators of disease-associated molecular alterations (4, 5, 6). Aberrant N-glycosylation has been implicated in numerous diseases, including cancer progression, immune dysregulation, metabolic disorders (7, 8, 9), highlighting the importance of site-specific N-glycopeptide analysis in both biological and clinical research (10, 11).

Recent advances in high-resolution mass spectrometry have dramatically increased analytical depth and acquisition speed, enabling large-scale cohort studies (6, 12, 13). However, overall throughput and reproducibility remain constrained by upstream sample preparation, particularly N-glycopeptide enrichment, which represents a rate-limiting step in glycoproteomics workflows (14).

A variety of enrichment strategies—including hydrophilic interaction chromatography (HILIC), lectin affinity purification, TiO_2_-based methods, mixed anion exchange (MAX) and strong anion exchange (15, 16, 17)—have been widely adopted to facilitate N-glycopeptide analysis.

While these approaches have contributed substantially to the field, integrating multiple sample preparation steps into a standardized and reproducible high-throughput workflow remains challenging, particularly in studies involving large sample numbers where analytical consistency is critical (14, 18, 19). To address these limitations, we developed a high-throughput and standardized enrichment workflow based on positive-pressure processing in a 96-well format. The workflow incorporates a 3 kDa molecular weight cut-off (MWCO) filtration step prior to MAX enrichment, enabling removal of low-molecular-weight contaminants while retaining most glycopeptides due to their increased molecular weight relative to most non-glycosylated peptides. Although MWCO filtration has been used primarily for desalting or cleanup (20), its integration into a global automated enrichment pipeline has not been systematically evaluated.

Blood-derived plasma and serum are the most commonly used samples for glycoprotein biomarker discovery due to their ease of sample collection and their rich representation of disease-associated glycosylation changes (21, 22, 23). However, immunodepletion of highly abundant serum proteins may introduce analytical bias (24, 25, 26) through potential co-depletion of interacting glycoproteins, particularly in site-specific glycoproteomic analyses (27). Therefore, glycoproteomic analysis of these biofluids requires highly reproducible, scalable, and depletion-free workflows capable of processing large sample cohorts while preserving glycoproteome integrity.

Here we present the 3kMAX workflow integrating MWCO-based pretreatment and MAX enrichment and demonstrate its analytical performance, reproducibility, and applicability to clinical serum samples, establishing a standardized platform for large-scale, site-specific glycoproteomics.

## Experimental Procedures

### Experimental Design and Statistical Rationale

The analytical performance and reproducibility of the 3kMAX workflow were evaluated using commercially available pooled human serum samples. For sensitivity and robustness assessment, 16 technical replicates were prepared for each serum input volume (5, 10, and 20 μl) and analyzed by single-shot–MS/MS. Quantitative reproducibility was assessed by calculating coefficients of variation (CVs) for all quantified glycopeptides. For method comparison, three enrichment strategies—3kMAX, manual MAX, and 3 kDa MWCO filtration alone—were evaluated using eight technical replicates per method under two acquisition schemes (25 and 45 samples per day). Identification depth, oxonium ion–containing MS/MS spectra, and overlap of identified glycopeptides were used as performance metrics. Clinical applicability was assessed using serum samples from normal controls and hepatocellular carcinoma (HCC) patients, processed with the optimized 10 μl serum input. All procedures involving human participants were approved by the Institutional Review Board of the National Cancer Center (IRB No. NCC2022-0355), and written informed consent was obtained from all participants in accordance with the Declaration of Helsinki. The demographic and clinical characteristics of the study participants are summarized in Supplemental Table S1. Differential glycopeptide abundance between groups was evaluated using two-sample Student’s t-tests (*p* < 0.05). Glycopeptide intensities were log_2_-transformed prior to statistical analysis, and features present in at least 60% of samples (*i.e.,* excluding features with more than 40% missing values) were retained for downstream analysis.

### Materials

Blood samples were collected using 5 ml serum separator tubes (gel type) and serum was aliquoted into 1.8 ml cryotubes or 0.75 ml 2D screw-cap tubes for storage. Pooled human serum (S1-M), urea (U5378), iodoacetamide (IAA; I1149), and 3 kDa (MWCO) ultra centrifugal filters (UFC5003) were purchased from Sigma-Aldrich (St Louis, MO). Bond-Breaker tris(2-carboxyethyl)phosphine (Cat. No. 77720), MS-grade trypsin (Cat. No. 90058), PepMap Neo trap cartridge (300 μm ID × 5 mm, 5 μm C18, 100 Å; Cat. No. 03-255-018), and formic acid (Optima LC–MS grade; Cat. No. A117-50) were obtained from Thermo Fisher Scientific (Waltham, MA). Oasis MAX 96-well plates (Cat. No. 186000373) and Sep-Pak tC18 96-well μElution plates (Cat. No. 186002318) were purchased from Waters Corporation (Milford, MA). HILIC SPE 96-well plates (Cat. No. 200.096.0025) were purchased from HILICON AB (Umeå, Sweden). Acetonitrile (ACN; LiChrosolv Hypergrade for LC–MS, Cat. No. 1.00029.1000) and LC–MS–grade water (LiChrosolv LC–MS grade, Cat. No. 1.15333.2500) were purchased from Merck (Darmstadt, Germany). An Aurora Ultimate C18 analytical column (25 cm × 75 μm, 1.7 μm C18; Cat. No. AUR2-25075C18) was obtained from IonOpticks (Melbourne, VIC, Australia).

### Blood Collection and Serum Processing

Blood samples were collected by trained medical personnel via venipuncture either in the outpatient clinic following a minimum 8-h fasting period or, for a subset of samples collected prior to June 10, 2020, in the operating room after induction of anesthesia. All samples were collected in 5 ml serum separator tubes (gel type containing a clot activator and gel separator, without the use of additional anticoagulants, preservatives, or protease inhibitors. Following collection, samples were allowed to clot and were processed for serum separation within 30 min to a maximum of 2 h. Serum was obtained by centrifugation at 3000 rpm for 10 to 15 min, with processing performed at temperatures up to 4 °C depending on the cohort. Immediately after centrifugation, the separated serum was aliquoted and stored at −80 °C. Aliquoting was performed using either 1.8 ml cryotubes (for samples collected up to 2021) or 0.75 ml 2D screw-cap tubes (from 2022 onwards). All serum samples were maintained at −80 °C until analysis. Following the first thaw for proteomics aliquoting, the samples were re-stored at −80 °C. The present analyses were subsequently performed using second-thaw specimens. No additional freeze–thaw cycles were applied prior to analysis. All samples were processed according to a standardized protocol; minor variations were limited to the collection setting (outpatient clinic versus operating room) and storage tube format depending on the year of acquisition.

### Protein Digestion on 3 kDa MWCO Filters

A 3 kDa MWCO filter unit (Millipore) was activated by adding 400 μl of LC-grade distilled water (DW) followed by centrifugation at 10,000×*g* for 30 min at 4 °C. The residual water in the filter was removed, and 5, 10 and 20 μl of intact serum was loaded. Subsequently, 40 μl of 8 M urea in 50 mM Tris-HCl (pH 8.0) was added, and the mixture was incubated at RT for 30 min. Reduction was performed by adding 10 μl of 100 mM tris(2-carboxyethyl)phosphine and incubating at RT for 30 min, followed by alkylation with 10 μl of 200 mM iodoacetamide (IAA) for 30 min at RT in the dark.

To reduce the urea concentration, 200 μl of 50 mM triethylammonium bicarbonate was added, and trypsin was added at a 1:50 (enzyme:protein) ratio. The sample was incubated at 37 °C for 18 h. Digestion was quenched by adding 3 μl of TFA. Small molecules and non-target peptides were removed by centrifugation at 10,000×*g* for 1 h at 4 °C. The filtrate collected in the filter reservoir was recovered for the desalting step.

### Protein Digestion by In-Solution Digestion

In-solution digestion was performed directly in the tube using 10 μl of intact serum. The subsequent reduction, alkylation, and tryptic digestion steps were carried out under the same conditions as described for the digestion protocol on 3 kDa MWCO filters.

### Automated Desalting of Peptides

For automated desalting using a positive pressure liquid handler system, Sep-Pak tC18 96-well plates (Waters) were sequentially conditioned with 0.4 ml of methanol (MeOH; twice), 400 μl of ACN (twice), and 600 μl of DW containing 0.1% TFA (v/v) (LC/MS grade; twice). Peptides obtained from the 3 kDa MWCO filter-based digestion and the in-solution digestion were loaded onto the conditioned Sep-Pak tC18 plates. Non-target components were removed by washing with 600 μl of DW containing 0.1% TFA (v/v) (twice). Finally, peptides were eluted with 600 μl of 80% ACN (v/v) containing 0.1% TFA (v/v) and collected for subsequent glycopeptide enrichment.

### High-Throughput Automated N-Glycopeptide Enrichment

To automatically enrich glycopeptides using a positive pressure liquid handler system, MAX 96-well plates (Waters) were sequentially conditioned with 1 ml of MeOH (twice), 1 ml of ACN (twice), 1 ml of 100 mM triethylammonium acetate (TEAA; three times), 1 ml of DW (LC/MS grade; five times), and 1 ml of 95% ACN (v/v) containing 1% TFA (v/v) (five times). The eluate obtained from the desalting step was supplemented with 600 μl of 98% ACN (v/v) containing 2% TFA (v/v) and loaded onto MAX 96-well plates. Non-glycopeptides were removed by washing with 1 ml 95% ACN (v/v) containing 1% TFA (v/v) (three times). Finally, glycopeptides were eluted with 600 μl of 50% ACN (v/v) containing 0.1% TFA (v/v), dried, and stored at −80 °C until LC–MS/MS analysis.

For HILIC enrichment, the iSPE-HILIC 96-well plates (Hilicon) were washed three times with 1 ml of 1% TFA solution, followed by addition of three 1 ml volumes of 80% ACN 1% TFA. One milligram of peptides and glycopeptides was added to 80% ACN and 1% TFA and the flow through was passed through the cartridge again. Samples were further washed with three 1 ml volumes of 80% ACN 1% TFA, eluted with 500 μl of 0.1% TFA, dried, and stored at −80 °C until MS analysis.

### LC-MS/MS Analysis

LC–MS/MS analyses were performed using an Orbitrap Astral Zoom MS prototype coupled to a Thermo Fisher Scientific Vanquish Neo Ultra High-Performance Liquid Chromatography system and interfaced via an EASY-Spray ion source. Peptides were separated on an Aurora Series C18 analytical column (25 cm length). Peptide separation was carried out using nano-flow reversed-phase liquid chromatography with 25 and 45 samples per day (SPD) gradient methods.

Data-dependent acquisition was performed with a fixed cycle time of 1 s. Full MS1 scans were acquired in the Orbitrap analyzer over an m/z range of 380 to 2000 at a resolution of 180,000, with an automatic gain control target of 5e6. Precursor ions were isolated using a quadrupole isolation window of 2 Th and fragmented by stepped higher-energy collisional dissociation (stepped HCD) with normalized collision energies of 24/32/40. MS/MS spectra were acquired in the Astral analyzer with an automatic gain control target of 1e5. For the 25 and 45 SPD methods, the active gradient times were 50 min and 30 min, respectively.

### Data Processing

The raw data files were analyzed using pGlyco3.1 software (28, 29, 30). For label-free samples, the parameters were set as follows: oxidation [M, +15.995 Da] and acetyl [ProteinN-term, +42.011 Da] for variable modifications. Carbamidomethyl [C, +57.022 Da] for fixed modification, maximum three missed cleavages, 10 ppm for precursor tolerance, 20 ppm for fragment tolerance, trypsin for enzyme, and a false discovery rate (FDR) of 1%. A curated human serum protein database was used for N-glycopeptide identification. The database was constructed by selecting proteins with blood concentration values greater than zero from the Human Protein Atlas (https://www.proteinatlas.org). The corresponding protein sequences were retrieved from UniProt (release: July 17, 2025), yielding a total of 4300 entries. The human N-glycan database in pGlyco3.1 was used.

For independent validation, raw data were re-analyzed using the FragPipe platform (v23.1; https://fragpipe.nesvilab.org) with the default 'glyco-N-HCD' workflow (31). Protein identification was performed against the same curated human serum protein database used for pGlyco analysis, which was constructed by selecting proteins with blood concentration values greater than zero from the Human Protein Atlas and retrieving the corresponding sequences from UniProt (release: July 17, 2025), yielding a total of 4300 entries. The database was appended with common contaminants and reversed decoy sequences. Enzymatic digestion settings and modifications remained consistent with the primary analysis: carbamidomethylation [C, +57.022 Da] as a fixed modification, and oxidation [M, +15.995 Da] and protein N-terminal acetylation [+42.011 Da] as variable modifications. The precursor and fragment ion mass tolerances were both set to 20 ppm. For N-glycopeptide identification, a mass-offset search strategy was employed using the same human N-glycan database as used in pGlyco3.1 (708 entries), with a glycan mass tolerance of 30 ppm. Identification results were strictly filtered to a 1% FDR at the glycopeptide level.

### Data Analysis

Identified intact N-glycopeptides were classified into glycan-type categories based on their monosaccharide compositions. N-glycans were classified into high-mannose (2 N-Acetyl-Hexosamine (HexNAc) and ≥5 Hexose (Hex)), complex/hybrid (≥3 HexNAc and ≥3 Hex), truncated (structures containing fewer than five HexNAc residues and lacking both fucose and sialic acid), and other types, according to the glycan annotations assigned by pGlyco3.1. Raw LC–MS/MS data were processed using pGlyco3.1 with a FDR of 1% at the glycopeptide level. Label-free quantification was performed using extracted ion chromatogram–based intensities of intact N-glycopeptides. All quantification in this study represents relative quantification rather than absolute quantification. For quantitative analyses, each intact glycopeptide, defined as a unique combination of peptide sequence and glycan composition, was treated as an independent feature, and intensities were aggregated only across replicate measurements of the same feature. Quantitative data processing, including calculation of averaged intensities and CV (%), was performed using Perseus (version 1.6.15.0) (32). Glycopeptides quantified in at least three technical replicates in at least one experimental condition were retained for downstream analysis. Intensities were log_2_-transformed, and missing values were imputed using a Perseus-style imputation strategy (32). Differential abundance analysis between experimental groups was performed using a two-sample Student’s *t* test, and resulting *p*-values were adjusted for multiple testing using the Benjamini–Hochberg FDR procedure. Adjusted *p*-values <0.05 were considered statistically significant. For multivariate analyses, normalized and log_2_-transformed intensities were used for principal component analysis (PCA) and hierarchical clustering. Gene ontology (GO) enrichment analysis of differentially abundant glycopeptides was conducted using Fisher’s exact test with a significance cutoff of *p* < 0.001 (33). Functional characterization of the up- and down-regulated differentially expressed glycoproteins was performed by GO enrichment analysis using Metascape (version v3.5.20260201), covering the biological process , molecular function, and cellular component categories (34). Data visualization and statistical plotting were performed using GraphPad Prism (version 9.5.1; https://www.graphpad.com).

## Results

### High-Throughput Automatic Glycopeptide Enrichment Workflow

**Fig. 1:**
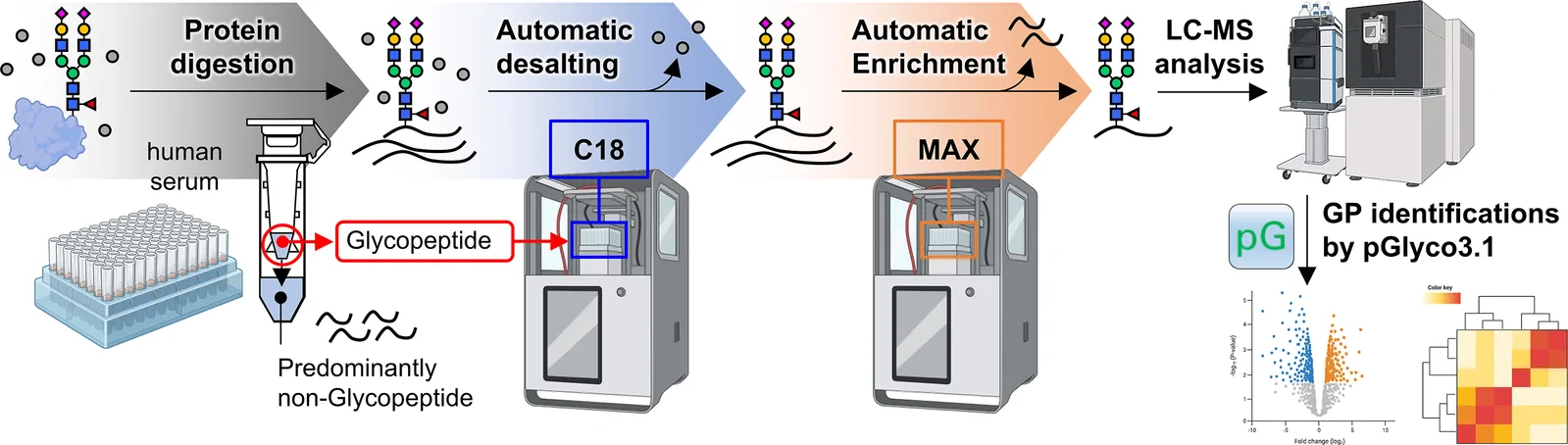
**Schematic scheme of the automatic glycopeptide enrichment method using 3k molecular weight cut-off filter and a positive pressure liquid handler system**.

An overview of the automated high-throughput N-glycopeptide enrichment workflow—integrating a 3 kDa MWCO filtration step with a positive pressure liquid handler system—is presented in Figure 1. Traditional enrichment approaches, including tip based HILIC, ZIC-HILIC, lectin affinity purification, TiO_2_-based methods, and strong anion exchange, have substantially advanced glycoproteomics. However, although these methods can be automated, integrating multi-step glycoproteomics sample preparation into a standardized and reproducible high-throughput workflow remains challenging. In contrast, our streamlined workflow combines MWCO-based prefractionation with automated glycopeptide enrichment, enabling robust and reproducible processing while reducing manual handling and processing time. The system processes up to 96 samples per run, with approximately 1 h required for desalting and 2 h for glycopeptide enrichment.

Previously, Yu Lin and colleagues demonstrated quantitative glycopeptide analysis of serum from HCC patients using LC–stepped-HCD–parallel reaction monitoring–MS/MS by incorporating a single additional step into the tryptic digestion workflow: enrichment and desalting with a 3 kDa MWCO filter (35). Although their study highlighted the reduced sample-handling steps and the simplicity of applying a 3 kDa MWCO filtration step, it did not report any performance improvements associated with MWCO-based enrichment. In our workflow, we hypothesized that introducing a 3 kDa MWCO step prior to glycopeptide enrichment would improve the efficiency of downstream affinity-based capture by reducing low-molecular-weight, non-glycosylated peptides and overall sample complexity. It should be noted that MWCO filtration does not result in complete separation, but rather acts as a size-based prefractionation step rather than a glycopeptide-specific enrichment. Moreover, because glycosylation increases peptide molecular weight, most glycopeptides typically exceed the 3 kDa cutoff. Accordingly, this filtration step facilitates preferential retention of glycopeptides in the filter reservoir due to their increased molecular weight rather than glycopeptide-specific interactions, as the separation is governed by size-based filtration. Building on this principle, we directly applied the MWCO-filtered retentate to our automated enrichment workflow, achieving preferential and reproducible glycopeptide enrichment in a high-throughput system, as shown in Figure 1.

### Rationale for Enrichment and Data Analysis Strategy

To further evaluate the performance of the 3kMAX workflow, we compared it with a conventional HILIC-based enrichment strategy using identical serum input conditions (10 μl, n = 8) (Supplemental Fig. S1). While most glycopeptides and glycoproteins identified by HILIC were also detected using MAX, the total number of identifications was substantially higher with MAX, including many additional unique species. This trend is consistent with previous reports demonstrating improved glycopeptide recovery using MAX-based enrichment (16). Notably, comparison of glycan-type distributions across HILIC-unique, MAX-unique, and shared glycopeptides revealed broadly similar profiles, without evidence of strong enrichment bias toward a specific glycan class (Supplemental Fig. S1*E*). In addition, analysis of fucosylation and sialylation patterns within complex- and hybrid-type glycopeptides showed comparable distributions across these groups (Supplemental Fig. S1*F*), suggesting that the increased identification depth achieved by MAX is not driven by selective enrichment of specific glycan features but rather reflects improved overall coverage. Based on these results, the MAX-based approach was selected for subsequent analyses.

Additionally, glycopeptide identifications were validated using an independent glycoproteomics search engine, showing substantial overlap between the two approaches (Supplemental Fig. S2). While each search engine identified a set of unique glycopeptides, a considerable proportion of identifications were shared, supporting the overall robustness of the dataset. This observation is consistent with previous studies reporting general agreement across different glycoproteomics search engines despite methodological differences (30). Notably, pGlyco employs a glycan-first strategy with multi-level quality control, which may result in a more conservative set of glycopeptide identifications compared to alternative search strategies.

### Sensitivity and Robustness Across Different Serum Input Volumes

To establish an optimal serum input volume, we evaluated the sensitivity and quantitative robustness of the automated 3kMAX workflow using different starting volumes. Human serum volumes of 5, 10, and 20 μl were processed using the complete 3kMAX workflow, with 16 technical replicates prepared for each condition (n = 16). An identical amount of enriched glycopeptides (200 ng) from all samples was analyzed by LC–stepped-HCD–MS/MS under the 25 SPD acquisition scheme.

**Fig. 2:**
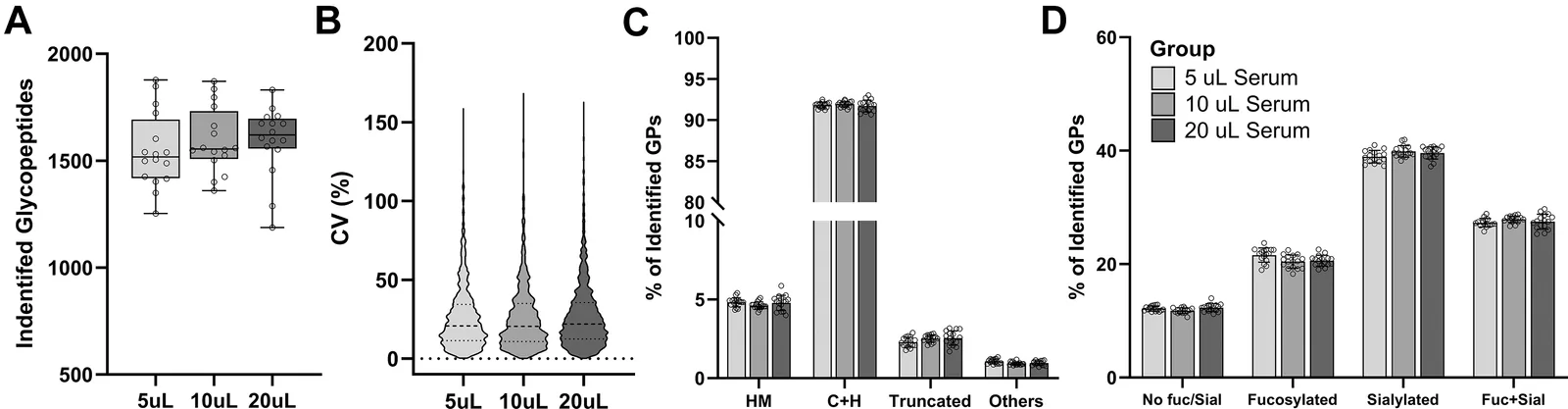
**Sensitivity assessment of the automated 3kMAX workflow using different human serum input volumes.***A,* numbers of identified unique glycopeptides obtained from 5, 10, and 20 μl sera (n = 16 each), with a normalized injection amount of 200 ng. *B, violin plots* showing the distributions of coefficients of variation (%) for quantified glycopeptides under each condition. *C,* glycan-type distribution of glycopeptides identified from each serum input volume, demonstrating consistent glycan-type coverage independent of starting material amount. *D,* percentage of fucosylated and sialylated glycopeptides in complex-/hybrid-type N-glycopeptides. HM, High-Mannose type; C + H, Complex and Hybrid type. MAX, mixed anion exchange.

Across the three starting serum volumes, comparable numbers of unique glycopeptides were identified (Fig. 2*A*). On average, 1548 ± 177, 1595 ± 153, and 1593 ± 164 unique glycopeptides were identified from 5, 10, and 20 μl serum inputs, respectively, indicating efficient glycopeptide recovery over a fourfold range of starting serum volume.

Quantitative reproducibility was assessed by calculating CV (%) for all quantified glycopeptides across the 16 technical replicates (Fig. 2*B*). All quantitative comparisons presented here are based on relative glycopeptide abundances derived from label-free intensity measurements. The median CV values were 20.81% (5 μl), 20.46% (10 μl), and 21.87% (20 μl), with largely overlapping interquartile ranges (5 μl: 11.41–34.37%, 10 μl: 10.86–34.88%, and 20 μl: 12.43–35.61%). Among the three starting serum volumes, the 10 μl input exhibited the lowest median CV and a slightly narrower distribution, reflecting an optimal balance between quantitative robustness and practical sample usage. In addition, the distribution of glycopeptide intensities was highly comparable across input volumes, with median log_2_ intensities of 30.11 (5 μl), 30.03 (10 μl), and 30.28 (20 μl), and largely overlapping interquartile ranges (5 μl: 27.78–33.38, 10 μl: 27.58–33.18, and 20 μl: 27.80–33.54) (Supplemental Fig. S3). Notably, CV showed a clear inverse relationship with glycopeptide intensity, with low-intensity glycopeptides exhibiting substantially higher variability, whereas high-intensity glycopeptides demonstrated improved quantitative reproducibility. These results indicate that the observed variability is primarily driven by low-abundance signals near the detection limit rather than systematic differences across input conditions.

Glycan-type distributions were also highly consistent across all serum volumes, with comparable proportions of high-mannose, complex/hybrid, truncated, and other glycan types observed for the 5, 10, and 20 μl serum inputs (Fig. 2*C*). In addition, the relative proportions of glycopeptides classified as non-fucosylated and non-sialylated, fucosylated only, sialylated only, and both fucosylated and sialylated were highly similar across all three input volumes (Fig. 2*D*), indicating that glycan profiles were not affected by the starting serum volumes.

Collectively, these results demonstrate that the 3kMAX workflow maintains stable identification performance, quantitative reproducibility, and glycopeptide compositional coverage across a wide range of serum input volumes. Based on these considerations, 10 μl of serum was selected as the standard input volume for all subsequent experiments.

### Reproducibility of the Automated 3kMAX Workflow

Using the optimized serum input volume of 10 μl, we evaluated the repeatability and day-to-day reproducibility of the automated glycopeptide enrichment workflow. To this end, 16 human serum samples were processed per day over three consecutive days under identical experimental conditions, yielding a total of 48 independently enriched preparations. This design enabled assessment of both within-run consistency and potential variability introduced by day-to-day processing throughout the entire workflow. In our study, enrichment efficiency was assessed as the proportion of MS/MS spectra containing diagnostic oxonium ions at m/z 138.05 (HexNAc fragment), 204.09 (HexNAc), 274.09 (Neu5Ac−H_2_O), 292.10 (Neu5Ac), and 366.14 (HexHexNAc), consistent with the oxonium ion–based glycan scoring strategy implemented in pGlyco3.1 (28).

**Fig. 3:**
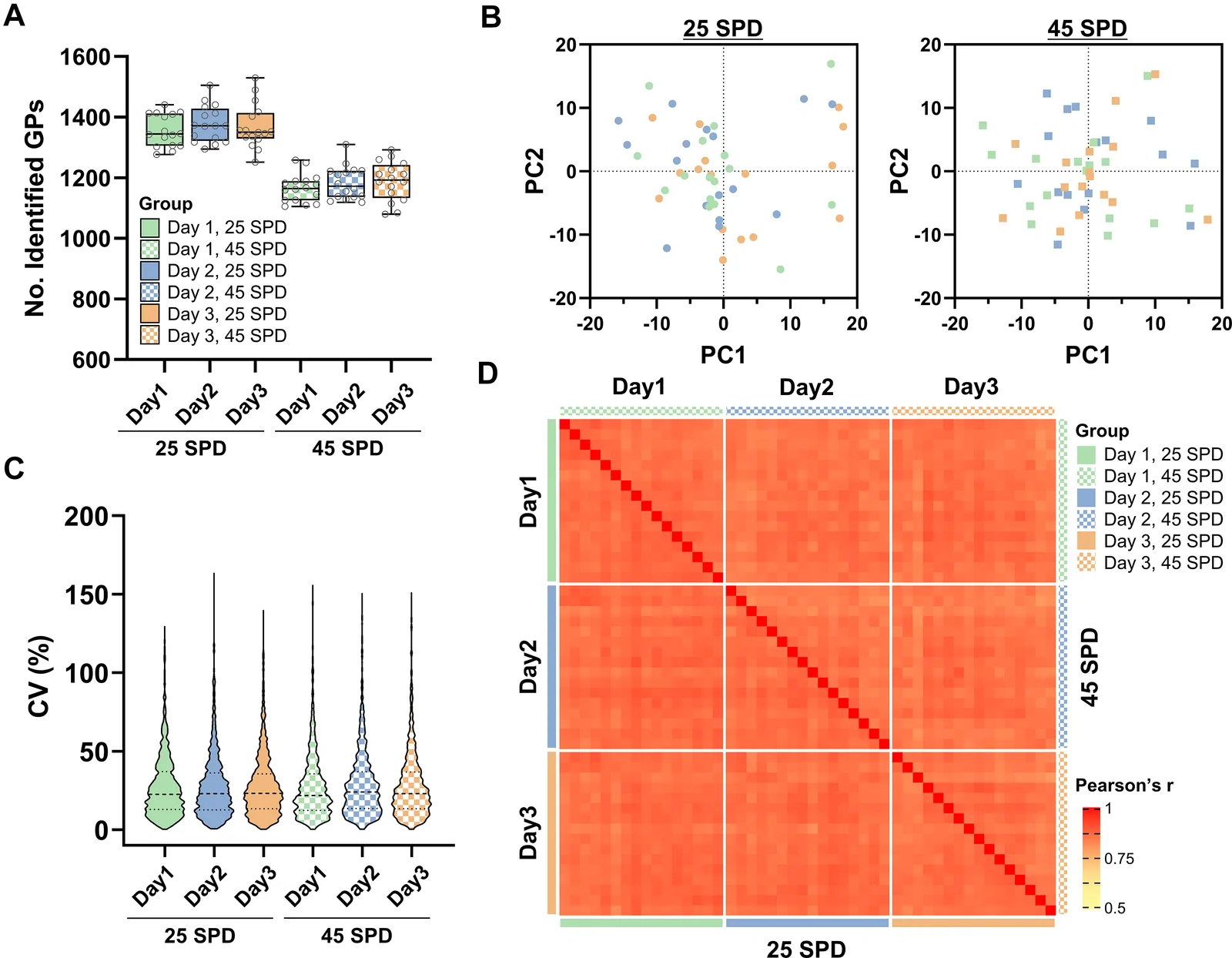
**Overview of glycopeptide identification reproducibility and data quality under different sampling densities.***A, box plots* showing the numbers of identified unique glycopeptides. *B,* principal component analysis plots of glycopeptide profiles acquired under 25SPD (*left*) and 45SPD (*right*) conditions. Each point represents one analysis, colored by experimental day (Day1–3). Data were filtered to include features detected in at least 60% of samples, log_10_-transformed, and processed using Perseus-style imputation. *C, violin plots* showing the distributions of coefficients of variation (%) for identified quantified glycopeptides under 25SPD and 45SPD conditions across 3 days (Day1–3). *Each box* and *violin plot* represents 16 analyses performed over three consecutive days for samples processed at 25 and 45 samples per day, respectively. *D,* correlation heatmap of glycopeptide intensities between the 25SPD and 45SPD datasets. Each cell represents the Pearson correlation coefficient between individual analyses across 3 days, with higher values (*red*) indicating strong agreement between conditions. SPD, samples per day.

Enrichment efficiency was assessed as the proportion of MS/MS spectra containing diagnostic oxonium ions. Across all three processing days, the oxonium ion rate remained consistently high, ranging from 92.97% to 96.37% (Supplemental Fig. S4), indicating stable enrichment selectivity and efficient recovery of bona fide N-glycopeptides with minimal day-dependent fluctuation. Subsequent LC–stepped-HCD–MS/MS analysis yielded an average of 1367 ± 62 unique glycopeptides under the 25 SPD condition and 1179 ± 55 under the 45 SPD condition, with only minor variation across processing days (Fig. 3*A*). The 25 SPD and 45 SPD methods correspond to chromatographic gradients of 50 min and 30 min, respectively. The longer gradient used in the 25 SPD condition provides improved chromatographic separation, resulting in increased identification depth compared to the shorter gradient. Although the absolute number of identifications differed between acquisition densities of 25 and 45 SPD, both throughput settings exhibited stable identification performance across days, supporting the robustness of the workflow under different MS sampling regimes.

Unsupervised PCA based on quantified glycopeptide intensities showed no clustering by processing day under either throughput condition (Fig. 3*B*), suggesting the absence of detectable batch effects and indicating that the workflow produces highly comparable quantitative glycoproteomic profiles across independent processing days. Quantitative variability was evaluated using the coefficient of variation (CV, %). CV distributions were highly similar across days and throughput settings, with median CVs ranging from 22.60 to 23.18% for 25 SPD and 21.76 to 24.01% for 45 SPD (Fig. 3*C*). Interquartile ranges were consistently maintained between approximately 12% and 37%, indicating stable quantitative behavior for the majority of glycopeptides.

Correlation analysis across all 48 enriched samples further supported high inter-sample consistency. Pearson correlation coefficients ranged from 0.698 to 0.842, with a mean off-diagonal correlation of 0.779 (Fig. 3*D*). Importantly, high correlation was maintained not only within the same processing day but also across days, demonstrating that the automated 3kMAX workflow provides reproducible enrichment and robust quantitative performance. Collectively, these results confirm that 3kMAX enables consistent, high-throughput serum glycoproteomic profiling across multiple processing days even with a low serum input volume of 10 μl.

### Enhanced Performance Compared with Conventional Methods

Having established the robustness and reproducibility of the 3kMAX workflow under standardized conditions (Fig. 3), we next evaluated its analytical performance in comparison with conventional glycopeptide enrichment strategies. Three workflows were assessed (1): 3 kDa MWCO filtration followed by automated MAX enrichment (3kMAX) (2), traditional manual MAX enrichment (MAX), and (3) 3 kDa MWCO filtration alone without subsequent enrichment (3k MWCO) (Supplemental Fig. S5).

**Fig. 4:**
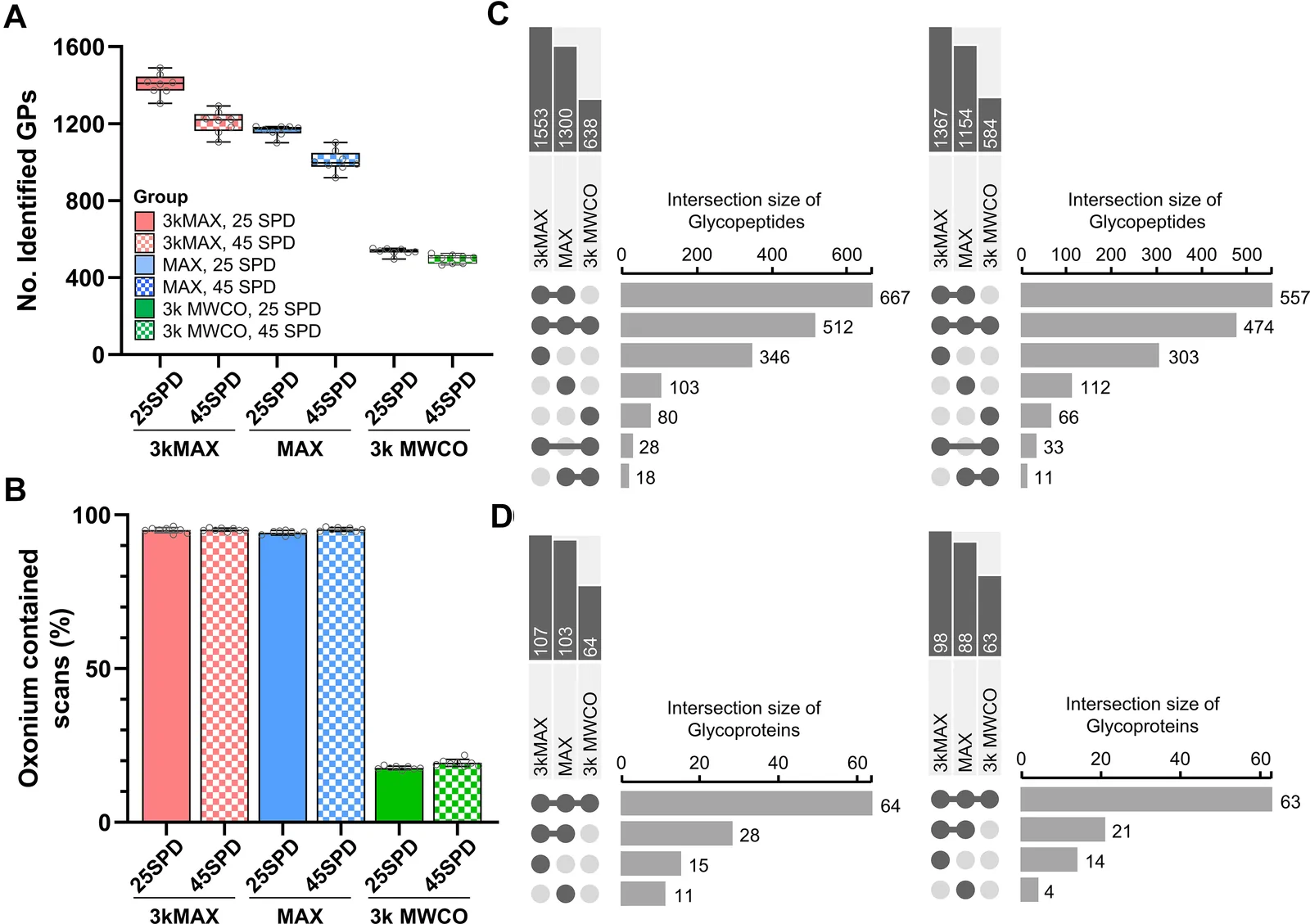
**Evaluation of glycopeptide enrichment performance using different sample preparation methods.***A, box plots* showing the numbers of identified unique glycopeptides obtained using three enrichment workflows: 3k molecular weight cut-off, MAX, and 3kMAX. *B, bar charts* showing the percentages of oxonium ion–containing scans from each method. *C and D,* UpSet plots illustrating the overlaps of identified glycoproteins *(C)* and glycopeptides *(D)* among the three preparation methods, with results obtained under the 25-SPD; *left* and 45-SPD; *right* acquisition schemes shown side by side. MAX, mixed anion exchange; SPD, samples per day.

Each workflow was applied to batches of eight technical replicates and analyzed under both 25 and 45 SPD acquisition schemes. Under both throughput conditions, the 3kMAX workflow consistently yielded the highest numbers of identified glycopeptides. Specifically, 3kMAX identified approximately 18 to 22% more unique glycopeptides than MAX, and 2.4–2.6-fold more glycopeptides than 3k MWCO alone, demonstrating a substantial improvement in analytical depth over conventional approaches (Fig. 4 and Table 1).

**Table 1:** Comparative analytical performance of three glycopeptide preparation methods under 25 and 45 SPD conditions

Analytical condition	Method	Total MS2 scan	Oxonium Contained Scans	Oxonium Contained Scans ratio (%)	No. Glycopeptide-spectrum match s	No. unique GPs
25 SPD	3kMAX	159,462 ± 3134	151,536 ± 3335	95.0	6556 ± 372	1404 ± 56
	MAX	159,365 ± 1864	149,914 ± 2667	94.1	5198 ± 252	1161 ± 28
	3k MWCO	396,824 ± 7419	70,243 ± 1680	17.7	1277 ± 59	535 ± 18
45 SPD	3kMAX	94,075 ± 2292	89,546 ± 2481	95.2	3930 ± 186	1207 ± 59
	MAX	92,557 ± 1313	88,163 ± 1105	95.3	3130 ± 232	1006 ± 55
	3k MWCO	226,204 ± 5386	43,678 ± 1664	19.3	981 ± 47	497 ± 23

Glycopeptide-spectrum match, number of identified unique glycopeptides, MS2 Scans and Oxonium contained scans ratio (%) were calculated using results obtained by pGlyco3.1.

To assess whether the observed differences in identification depth were associated with glycan compositional bias, we compared the structural characteristics of glycopeptides identified by each preprocessing workflow using the total glycopeptide pools from the 25 SPD and 45 SPD acquisition schemes, respectively (Supplemental Fig. S6*A*). Across both acquisition schemes, complex/hybrid-type N-glycopeptides constituted the dominant structural class for all workflows, whereas high-mannose-type glycopeptides consistently represented a minor fraction. Although the overall glycan-class composition was broadly conserved, subtle workflow-dependent shifts were observed: the 3kMAX and MAX workflows showed relatively higher contributions from high-mannose-type glycopeptides compared with the 3k MWCO workflow, and 3kMAX consistently exhibited the highest proportion of complex/hybrid-type glycopeptides. Truncated and other glycan classes remained measurable but low across all conditions.

Similarly, analysis of fucosylation and sialylation patterns within complex- and hybrid-type glycopeptides—calculated relative to the total glycopeptide pool for each workflow—revealed no evidence of systematic enrichment toward a single modification state (Supplemental Fig. S6*B*). Nevertheless, the overall abundance of complex/hybrid-type glycopeptides across all modification categories (non-fucosylated/non-sialylated, fucosylated, sialylated, and doubly modified species) was consistently higher in the 3kMAX workflow compared with MAX and 3k MWCO. In particular, 3kMAX showed elevated levels of sialylated and doubly modified (fucosylated + sialylated) glycopeptides, suggesting improved recovery of structurally diverse complex/hybrid-type glycopeptides rather than selective enrichment of a specific glycan feature. Collectively, these results indicate that the enhanced performance of the 3kMAX workflow is primarily driven by increased overall coverage of complex/hybrid-type glycopeptides while maintaining broadly comparable glycan structural distributions across workflows.

To further characterize the analytical features of method-specific glycopeptides, we examined the physicochemical and quantitative properties of glycopeptides uniquely identified by each workflow (Supplemental Fig. S7). Analysis of glycan-type distribution and modification patterns in the method-specific glycopeptide sets revealed broadly comparable profiles across workflows, with no evidence of strong compositional bias (Supplemental Fig. S7, *A* and *B*). Scatter plot analyses of molecular weight versus abundance revealed that method-specific glycopeptides generally exhibited lower abundances compared with the overall glycopeptide population, consistent with the detection of features near the analytical sensitivity limit (Supplemental Fig. S7*C*). Notably, glycopeptides uniquely identified by the 3kMAX workflow spanned a broad range of molecular weights (2-7 kDa) under both 25 and 45 SPD conditions, indicating expanded coverage of glycopeptide feature space relative to either MAX or MWCO alone. In contrast, glycopeptides uniquely identified by the 3k MWCO workflow showed a comparatively restricted distribution, consistent with the size-selective nature of filtration-based enrichment. Similarly, analysis of the MWCO filtrate fraction revealed a marked reduction in glycopeptide-spectrum matches (Supplemental Fig. S8*A*), as well as in the number of identified glycopeptides (Supplemental Fig. S8*B*) and glycoproteins (Supplemental Fig. S8*C*), compared to the retentate fraction. In addition, comparison of glycopeptide molecular weight distributions showed a clear shift toward lower molecular weight species in the filtrate (median ∼3.0 kDa) relative to the retentate (median ∼4.2 kDa) (Supplemental Fig. S8*D*). These results indicate that while glycan-related signals may be present in the filtrate, intact glycopeptides are predominantly retained during MWCO filtration.

Moreover, glycopeptides uniquely identified by the 3kMAX workflow exhibited higher detection frequencies compared with those identified by MAX or 3k MWCO alone (Supplemental Fig. S7*D*). Across both throughput conditions, the 3kMAX workflow showed the highest number of low-abundance glycopeptides (log_2_ abundance <25), supporting improved analytical sensitivity and robustness.

Collectively, these results demonstrate that integrating 3 kDa MWCO–based pre-filtration with automated MAX enrichment not only increases glycopeptide identification depth and selectivity but also expands the accessible glycopeptide feature space while maintaining reproducible detection across different acquisition throughputs.

### Application to Clinical Serum Glycoproteomics

To evaluate the applicability of the 3kMAX workflow to clinical serum samples, quantitative glycoproteomic profiling was performed using sera from normal individuals (n = 23) and patients with HCC (n = 24). The demographic and clinical characteristics of the study participants are summarized in Supplemental Table S1. Across all samples, a large number of glycopeptides were consistently identified, with an average of 1471 glycopeptides detected in normal serum samples and 1273 glycopeptides detected in HCC serum samples (Supplemental Fig. S9*A* and Supplemental Table S2). High proportions of oxonium ion–containing MS/MS spectra further supported robust glycopeptide enrichment and confident identification across the cohort (Supplemental Fig. S9*B*).

**Fig. 5:**
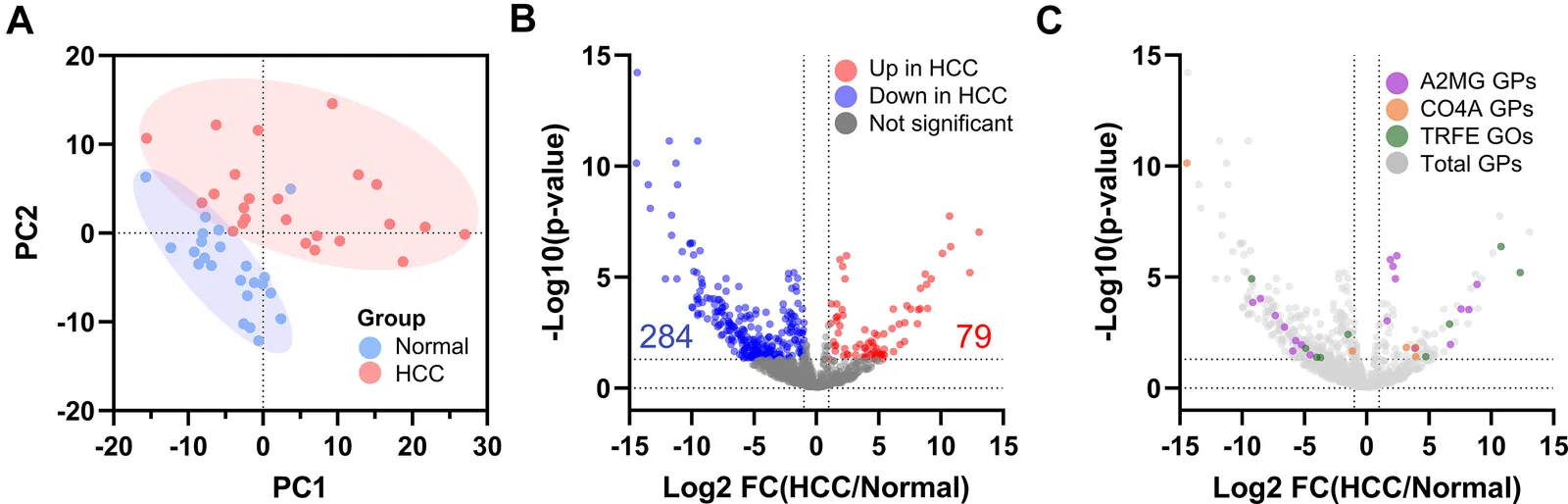
**Quantitative glycoproteomic analysis of normal and HCC serum samples.***A,* principal component analysis of quantified serum glycopeptides from normal (n = 23) and hepatocellular carcinoma (HCC; n = 24) samples. *B,* volcano plot illustrating differential expression patterns of identified glycopeptides between HCC and normal serum samples. The x-axis represents the log2 fold change (HCC *versus* normal), and the y-axis represents the −log10 adjusted *p*-value. Glycopeptides significantly up-regulated in HCC are shown in *red*, those down-regulated in HCC are shown in *blue*, and nonsignificant features are shown in *gray*. *C*, volcano plot highlighting glycopeptides showing opposite regulation patterns (both up- and down-regulation) within the same protein between HCC and normal serum samples. HCC, hepatocellular carcinoma.

Unsupervised PCA based on quantified glycopeptide intensities revealed a clear separation between normal and HCC serum samples, indicating distinct glycoproteomic profiles associated with disease status (Fig. 5*A*). Hierarchical clustering of differentially expressed glycopeptides further demonstrated grouping of samples according to clinical classification, with changes in glycopeptide abundance observed across patient cohorts (Supplemental Fig. S9*C*).

Differential expression analysis identified a subset of glycopeptides that were significantly altered between HCC and normal sera (Fig. 5*B*). Among these, 79 glycopeptides were significantly up-regulated and 284 glycopeptides were significantly down-regulated in HCC relative to normal controls. These results indicate remodeling of the serum glycoproteome in HCC at the glycopeptide level.

To assess whether the observed abundance changes reflect biological processes previously implicated in liver disease–associated glycosylation alterations, functional enrichment analysis was performed on glycopeptides significantly up-regulated in HCC (Supplemental Fig. S9*D*). The up-regulated glycopeptides were enriched in GO terms related to acute-phase response, complement and coagulation cascades, and immune-associated processes. These functional trends are consistent with glycosylation-associated pathways reported in prior studies of liver disease progression (36).

Importantly, beyond global changes in glycopeptide abundance, the dataset also revealed evidence of site-specific regulation. Specifically, several proteins contained both significantly up- and down-regulated glycopeptides within the same protein context, suggesting that glycosylation remodeling may occur in a site-specific manner. To highlight this phenomenon and motivate further site-resolved interpretation, glycopeptides exhibiting opposing regulation within the same protein were annotated on the volcano plot (Fig. 5*C*), with representative examples including α-2-macroglobulin (A2MG), complement C4-A, and transferrin (TRFE).

Together, the results from Figure 5 and Supplemental Fig. S9 demonstrate that the 3kMAX workflow enables robust and reproducible quantitative glycoproteomic profiling of clinical serum samples and captures disease-associated glycopeptide-level alterations that are biologically coherent with existing literature. These findings support the analytical validity of the workflow for large-scale clinical glycoproteomics rather than serving as evidence for biomarker validation.

### Site-dependent N-Glycosylation Changes in HCC Serum

Motivated by the observation that several proteins contained both significantly up- and down-regulated glycopeptides (Fig. 5*C*), we next examined the clinical serum glycoproteomic dataset from a site-specific N-glycoproteome perspective. Because disease-associated alterations in protein glycosylation often occur in a site-dependent manner rather than uniformly across an entire protein (37, 38), this analysis aimed to assess whether individual N-glycosylation sites within the same protein could be independently quantified and exhibit distinct regulatory patterns in HCC.

**Fig. 6:**
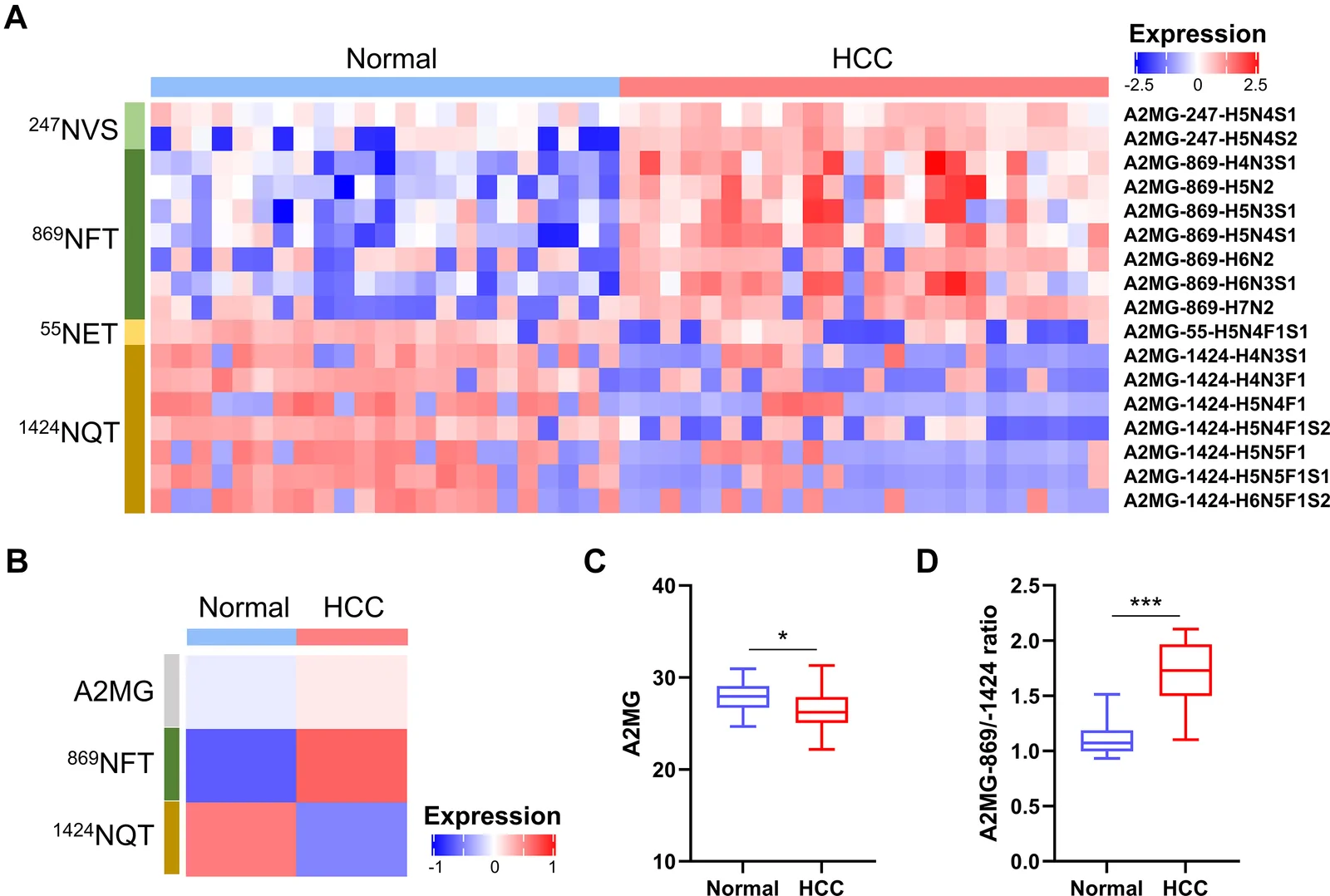
**Site-specific glycan profiling of A2MG identified in normal and HCC.***A,* Heatmap showing all the identified glycans at the glycosite Asn-55, Asn-869, and Asn-1424 of A2MG. *B*, Heatmap summarizing site-specific glycan profiles of A2MG, grouped by A2MG protein level, the 869NFT glycosite, and the 1424NQT glycosite. Values are displayed as z-scores. *C*, *box plot* comparing the A2MG protein level between normal and HCC samples, calculated using intensity values aggregated across all identified glycosites. *D*, *box plot* showing the ratio of glycan intensity between the 869NFT and 1424NQT glycosites (869/1424) for A2MG in normal and HCC samples. Statistical significance was determined by Student’s *t* test. *p* < 0.05 (∗), *p* < 0.01 (∗∗), and *p* < 0.001 (∗∗∗).A2MG, α-2-macroglobulin; HCC, hepatocellular carcinoma.

A2MG—a highly abundant liver-derived plasma glycoprotein (39, 40)—was selected as a representative exemplar due to its consistent detection across samples and the presence of multiple annotated N-glycosylation sites. Site-resolved visualization of A2MG revealed that glycopeptide signals originating from individual glycosites displayed pronounced divergence between normal and HCC serum samples (Fig. 6*A*): the 869NFT glycosite exhibited an overall increase in HCC, whereas the 1424NQT glycosite showed an opposing trend, indicating strong site-dependent remodeling that cannot be explained by protein-level abundance changes alone. Representative annotated MS/MS spectra supporting confident site-specific glycopeptide identification and glycosite assignment are provided in Supplemental Fig. S10. The overall A2MG protein level was calculated as the summed abundance of all quantified glycopeptides (Fig. 6*B*).

To quantitatively summarize this non-uniform regulation within a single protein, we calculated the ratio of total glycan-associated intensity between the 869NFT and 1424NQT glycosites (869/1424) for each sample. This site-to-site ratio showed a marked increase in HCC relative to normal sera (Fig. 6*D*), providing clearer discrimination than comparisons based on overall A2MG protein-level abundance.

In addition, an analogous site-specific analysis of TRFE reproduced the same overall phenomenon, demonstrating that the observed non-uniform, site-dependent glycosylation regulation is not unique to A2MG (Supplemental Fig. S11). Together, these results demonstrate that the 3kMAX workflow enables robust site-specific N-glycoproteomic quantification in clinical serum samples and captures glycosylation alterations that would be obscured by protein-level summaries.

As additional examples, we examined several glycoproteins that were consistently up-regulated in HCC. Site-level heatmaps for complement C3 (CO3), immunoglobulin heavy constant gamma 4, lumican, hepatocyte growth factor-like protein, and plasma protease C1 inhibitor showed coordinated increases across selected glycosylation sites, and representative intact glycopeptides were further summarized by box plots (Supplemental Fig. S12).

Collectively, these supplementary examples demonstrate that the 3kMAX workflow enables robust site-specific quantification not only for proteins exhibiting divergent intra-protein regulation, such as A2MG and TRFE, but also for up-regulated glycoproteins showing coordinated site-level increases, thereby supporting the general applicability of the approach to site-specific glycoproteomic analysis in clinical serum samples.

## Discussion

In this study, we present 3kMAX, an automated and depletion-free N-glycopeptide enrichment workflow that integrates 3 kDa MWCO–based filtration with positive-pressure–driven MAX enrichment in a 96-well format. By addressing the enrichment step—one of the primary bottlenecks in glycoproteomics—this workflow enables robust, reproducible, and high-throughput serum glycoproteomic analysis that is well aligned with the capabilities of modern mass spectrometry platforms. Importantly, by avoiding immunodepletion, the workflow minimizes the potential risk of codepleting glycoproteins associated with high-abundance carriers, an effect that has been reported in depletion-based proteomic workflows. This design helps preserve the native glycoproteome composition for site-specific quantitative analysis. It should be noted that the present study is based on relative quantification, which is suitable for comparative glycoproteomic analyses, whereas absolute quantification would require standardized reference materials and is beyond the scope of this work.

A key feature of the 3kMAX workflow is the strategic incorporation of MWCO-based filtration prior to automated MAX enrichment. Although MWCO filtration has traditionally been employed mainly for desalting or simple cleanup, our results demonstrate that its integration into a global enrichment workflow substantially improves N-glycopeptide selectivity and identification depth compared with either MAX enrichment or MWCO filtration alone. Importantly, this improvement was achieved without introducing detectable bias toward specific glycan structural classes or glycosylation features. When glycan-type composition and fucosylation/sialylation patterns were evaluated using the total glycopeptide pool obtained from each preprocessing workflow, overall distributions remained broadly consistent across methods (Supplemental Fig. S7, *A* and *B*). Although subtle workflow-dependent shifts were observed—such as relatively higher high-mannose contributions in 3kMAX and MAX and an overall higher representation of complex/hybrid-type glycopeptides in 3kMAX—these differences did not indicate selective enrichment of rare glycan categories. The consistently high proportion of oxonium ion–containing MS/MS spectra further supports efficient enrichment of bona fide N-glycopeptides by the 3kMAX strategy.

The automated nature of the 3kMAX platform represents a major practical advantage for large-scale studies. Parallel processing of up to 96 samples with minimal operator intervention markedly reduces hands-on time and inter-operator variability, which are common sources of irreproducibility in conventional glycoproteomics workflows. Benchmarking under different acquisition densities (25 and 45 SPD) demonstrated stable identification performance and quantitative reproducibility across multiple processing days, indicating that the workflow is robust to variations in MS throughput. The high scan speed and parallelized acquisition of the Orbitrap Astral platform enable dense MS/MS sampling, particularly under 25 and 45 SPD conditions. The automated 3kMAX workflow complements this capability by ensuring that sample preparation throughput and reproducibility are not limiting factors. In addition, sensitivity analysis across a fourfold range of serum input volumes identified 10 μl as an optimal compromise between analytical depth, quantitative robustness, and practical sample usage, supporting its suitability for clinical serum studies where sample availability is often limited.

Beyond improvements in throughput and robustness, feature-level analyses provided important insights into the analytical behavior of method-specific glycopeptides. Glycopeptides uniquely identified by the 3kMAX workflow generally exhibited lower abundance compared with the overall glycopeptide population, consistent with the detection of features near the analytical sensitivity limit. Notably, these features spanned a broad range of glycopeptide molecular weights and showed higher detection frequency across technical replicates than those uniquely identified by MAX or MWCO alone. This combination of expanded feature space coverage and reproducible detection indicates that the additional identifications achieved by 3kMAX reflect genuine analytical gains rather than stochastic or noise-driven effects.

Importantly, the preservation of glycopeptides across a wide molecular weight range in the 3kMAX workflow contrasts with the more pronounced size-dependent selectivity observed for MWCO filtration alone. This suggests that, in the integrated workflow, MWCO filtration primarily functions as a pre-cleanup step to reduce low-molecular-weight background components, whereas glycopeptide selectivity and recovery are largely governed by the subsequent MAX enrichment. As a result, size-dependent losses introduced during filtration are limited under the integrated workflow, enabling balanced recovery of both smaller and larger glycopeptides while maintaining reproducible detection. Together, these observations underscore the complementary roles of MWCO filtration and MAX enrichment in the 3kMAX workflow and explain its ability to achieve increased identification depth without introducing substantial compositional distortion.

Application of the 3kMAX workflow to hepatocellular carcinoma serum samples further highlighted the value of site-specific N-glycoproteomic analysis. Disease-associated remodeling of the serum glycoproteome was readily detected at the glycopeptide level (37, 38), and several proteins contained both significantly up- and down-regulated glycopeptides, implying non-uniform regulation across glycosylation sites. Site-specific analysis of A2MG—a highly abundant liver-derived plasma glycoprotein (39, 40)—demonstrated that overall protein-level abundance remained largely unchanged between normal and HCC sera, whereas individual glycosites exhibited pronounced divergence. In particular, the 869NFT and 1424NQT glycosites showed opposing trends, and the site-to-site ratio (869/1424) provided a robust quantitative summary that was markedly increased in HCC. A2MG is a major liver-derived acute-phase glycoprotein, and its glycosylation is closely linked to hepatic functional status (41). Given that HCC is associated with remodeling of complex-type N-glycans (42, 43, 44), the opposing regulation observed at the 869NFT and 1424NQT glycosites may reflect site-dependent enzymatic processing rather than uniform protein-level changes. Importantly, an analogous analysis of TRFE reproduced the same overall phenomenon, indicating that this site-dependent remodeling is not unique to a single protein. Transferrin glycosylation is known to be highly sensitive to hepatic functional status, and site-dependent remodeling may influence iron-binding stability or circulatory half-life (45). The observed divergence between individual glycosites further supports the notion that HCC-associated glycosylation changes are spatially regulated rather than uniformly distributed across the protein backbone. Supplementary analyses of additional up-regulated glycoproteins further confirmed that the workflow can reproducibly resolve coordinated site-level glycosylation changes across multiple proteins. Collectively, these results demonstrate that 3kMAX enables clinically scalable, site-resolved quantitative glycoproteomics and captures biologically coherent glycosylation remodeling that would be obscured by protein-level summaries or glycomics approaches lacking site-specific context.

Several limitations of the present study should be acknowledged. Although the observed site-specific glycosylation patterns are analytically robust and consistent with biological expectations, validation in larger and independent clinical cohorts will be required to establish their generalizability and potential utility as disease biomarkers. In addition, integration with complementary proteomic measurements may further clarify the relationship between protein abundance and site-specific glycosylation changes. Nevertheless, these considerations do not detract from the primary contribution of this work, which is the development and systematic validation of a scalable, reproducible, and high-throughput N-glycopeptide enrichment platform.

In summary, the 3kMAX workflow provides a practical and robust solution for large-scale, site-specific glycoproteomic profiling of serum samples. By combining automated processing, improved enrichment selectivity, and compatibility with high-throughput MS acquisition, this approach facilitates comprehensive and reproducible interrogation of disease-associated glycosylation changes and offers a solid foundation for future biomarker discovery and translational glycoproteomics applications.

## Conclusion

In this study, we developed 3kMAX, a depletion-free and high-throughput N-glycopeptide enrichment workflow that combines 3 kDa MWCO–based filtration with positive-pressure–driven MAX enrichment in a 96-well format. By addressing the enrichment step as a major bottleneck in glycoproteomics, the 3kMAX platform enables parallel processing of up to 96 serum samples with minimal manual intervention, substantially improving analytical throughput, reproducibility, and robustness.

Systematic benchmarking demonstrated that 3kMAX provides significantly enhanced glycopeptide selectivity and identification depth compared with conventional MAX enrichment or MWCO filtration alone, while maintaining broadly consistent glycan-class composition across workflows. The workflow maintained stable performance across different serum input volumes and acquisition densities, supporting the use of 10 μl serum as a practical and standardized input for clinical studies.

Application to hepatocellular carcinoma serum samples further demonstrated the utility of the platform for large-scale quantitative glycoproteomics and site-specific N-glycoproteome analysis. In addition to detecting extensive glycopeptide-level remodeling, site-resolved quantification revealed that individual N-glycosylation sites within the same protein can be differentially regulated even when overall protein abundance is unchanged. The A2MG exemplar highlighted pronounced divergence between glycosites, quantitatively summarized by a markedly increased 869/1424 site ratio in HCC, and a similar phenomenon was reproduced for TRFE. Collectively, these results establish 3kMAX as a scalable and robust foundation for high-throughput, site-specific serum glycoproteomics and provide a practical framework for future biomarker-oriented and translational glycoproteomic studies.

## Data Availability

The mass spectrometry proteomics data have been deposited to the ProteomeXchange Consortium (http://proteomecentral.proteomexchange.org) via the PRIDE partner repository (46, 47, 48) with the dataset identifier PXD074428 and are publicly available. The data are also accessible through the MassIVE repository under accession number MSV000100835.

## Supplemental data

This article contains supplemental data.

## Conflict of Interest

The authors declare no competing interests.
