# Supplementary material for: Depletion-free Automated Enrichment of Serum Glycopeptides for High-Throughput Clinical Glycoproteomics
Source: Mol Cell Proteomics. 2026 Jun 25;25(9):101611. doi: 10.1016/j.mcpro.2026.101611 (PMC13571447; doi:10.1016/j.mcpro.2026.101611)
Supplement: Supplementary file 1 [file mmc1.docx]

**Supporting information**

**Table 1** Characteristics of 47 participants clinical data of glycoproteomics.

| Characteristics | Normal (n=23) | HCC (n=24) |
| --- | --- | --- |
| Gender |  |  |
| Male | 13 | 20 |
| Female | 10 | 4 |
| Age (years) |  |  |
| Mean±SD | 66.50±13.61 | 59.54±11.42 |
| Etiology |  |  |
| NBNC | 23 | 10 |
| HBV | / | 12 |
| HCV | / | 2 |
