# Supplementary material for: Depletion-free Automated Enrichment of Serum Glycopeptides for High-Throughput Clinical Glycoproteomics
Source: Mol Cell Proteomics. 2026 Jun 25;25(9):101611. doi: 10.1016/j.mcpro.2026.101611 (PMC13571447; doi:10.1016/j.mcpro.2026.101611)
Supplement: Supplementary file 2 [file mmc3.docx]

**Supplemental figure S1-S12**

**Depletion-Free Automated Enrichment of Serum Glycopeptides for High-Throughput Clinical Glycoproteomics**

Jung Hoon Choi^1,2,†^, Geul Bang^1,†^, Jong Hyun Seol^1^, Seong Woo Choi^3^, Su-Min Lee^1^, Bao Tan Nguyen^1^, Seungjin Na^1,4^, Cheol-Jung Lee^1,4^, Heeyoun Hwang^5^, Jinsoo Chung^6^, Sang-Jae Park^7^, Sang Myoung Woo^7^, Hye Gwang Jeong^2,*^ and Jin Young Kim^1,8,9,*^

^1^Center for Target-to-Therapeutics Research, Korea Basic Science Institute (KBSI), Ochang 28119, Republic of Korea

^2^College of Pharmacy, Chungnam National University, Daejeon 34134, Republic of Korea

^3^Department of Bio and Brain Engineering, Korea Advanced Institute of Science and Technology (KAIST), Daejeon 34141, Republic of Korea

^4^University of Science and Technology (UST), Daejeon 34113, Republic of Korea

^5^Research Unit for Molecular Structure and AI Data, Korea Basic Science Institute (KBSI), Ochang 28119, Republic of Korea

^6^Center for Urologic Cancer, National Cancer Center, Goyang 10408, Republic of Korea

^7^Center for Liver and Pancreatobiliary Cancer, National Cancer Center, Goyang 10408, Republic of Korea

^8^Critical Diseases Diagnostics Convergence Research Center, Korea Research Institute of Bioscience and Biotechnology (KRIBB), Daejeon 34141, Republic of Korea

^9^Chung-Ang University College of Pharmacy, Seoul 06934, Republic of Korea.

^†^These authors contributed equally to this work.

**^*^For correspondence:** Jin Young Kim ([jinyoung@kbsi.re.kr](mailto:jinyoung@kbsi.re.kr)) and Hye Gwang Jeong ([hgjeong@cnu.ac.kr](mailto:hgjeong@cnu.ac.kr).)

**Supplemental figure S1. Comparison of glycopeptide identification performance between HILIC and MAX enrichment methods.** *A-B,* Bar charts showing the number of identified glycopeptides *(A)* and identified glycoproteins *(B)*. *C-D*, Venn diagrams showing the overlap of identified glycopeptides *(C)* and identified glycoproteins *(D)* between the two enrichment methods. Each dot represents an individual replicate. *E,* Glycan-type distribution of glycopeptides identified by the HILIC and MAX workflows, shown as proportions of total identified glycopeptides under identical acquisition conditions. *F-G*, Numbers of glycopeptides belonging to each glycan category within the complement set (HILIC-unique, MAX-unique, and shared features). *F,* Distribution of glycan structural classes. *G,* Numbers of fucosylated and sialylated glycopeptides among complex- and hybrid-type N-glycopeptides.

**Supplemental figure S2. Comparison of glycopeptide identification results obtained using pGlyco3.1 and MSFragger-Glyco.** *A,* Bar charts showing the number of identified glycopeptides and glycoproteins from HILIC and MAX enrichment workflows, analyzed using pGlyco3.1 and MSFragger-Glyco. Each dot represents an individual replicate. *B,* Glycan-type distribution of identified glycopeptides between pGlyco3.1 and MSFragger-Glyco for HILIC and MAX datasets. *C,* Percentage of fucosylated and sialylated glycopeptides in complex-/hybrid-type N-glycopeptides. HM, High-Mannose type; C+H, Complex and Hybrid type.

**Supplemental figure S3. Relationship between glycopeptide intensity and coefficient of variation (CV).** Serum input conditions were 5 µL (A), 10 µL (B), and 20 µL (C). Scatter plots show the relationship between log₂-transformed glycopeptide intensity and CV (%).

**Supplemental figure S4.** Bar charts representing the percentages of oxonium ion–containing scans. Each box and bar correspond to 16 analyses performed over three consecutive days for samples processed at 25 and 45 SPD, respectively.

**Supplemental figure S5. Schematic overview of the three glycopeptide preparation strategies compared in this study**. (1) 3kMAX workflow: tryptic digestion followed by 3 kDa MWCO filtration to retain glycopeptides, followed by automated glycopeptide enrichment on a positive pressure liquid handler system. (2) MAX workflow: traditional manual glycopeptide enrichment using MAX 96 well plates following tryptic digestion. (3) 3k MWCO workflow: tryptic digestion followed solely by 3 kDa MWCO filtration without subsequent enrichment. Colored boxes denote the three preparation schemes (red, blue, and green), and arrows indicate processing steps from digestion to LC–stepped-HCD–MS/MS analysis. The automated enrichment path highlights reduced manual handling and improved workflow reproducibility.

**Supplemental figure S6. Evaluation of glycopeptide patterns enriched by 3kMAX, MAX, and 3k MWCO methods.** *A,* Distribution of N-glycan types enriched by 3kMAX, MAX, and 3k MWCO methods. Minor differences in all type N-glycans were observed. *B,* Percentage of fucosylated and sialylated glycopeptides in complex-/hybrid-type N-glycopeptides. HM, High-Mannose type; C+H, Complex and Hybrid type.

**Supplemental figure S7. Evaluation of method-specific glycopeptide characteristics enriched by the 3kMAX, MAX, and 3k MWCO workflows.** *A*, Glycan-type distribution of glycopeptides in the complement set, shown as proportions of total glycopeptides identified by each workflow under identical acquisition conditions. *B*, Proportions of fucosylated and sialylated glycopeptides in the complement set among complex- and hybrid-type N-glycopeptides, normalized to the total glycopeptides identified by each method. *C,* Distribution of glycopeptide molecular weight (or peptide backbone length) of method-specific glycopeptides, illustrating differences in the physicochemical feature space captured by each workflow, with results obtained under the 25 samples-per-day (25-SPD; top) and 45 samples-per-day (45-SPD; bottom) acquisition schemes shown. Dotted lines indicate the median values for each distribution. *D,* Detection frequency of method-specific glycopeptides across technical replicates, indicating differences in reproducibility and analytical robustness among the workflows, with results from the 25-SPD (left) and 45-SPD (right) conditions displayed.HM, high-mannose type; C+H, complex and hybrid type.

**Supplemental figure S8. Comparison of glycopeptide-related metrics between 3 kDa MWCO retentate and filtrate fractions.** *A-C*, Bar charts showing the number of glycopeptide-spectrum matches (GSMs) *(A)*, the number of identified glycopeptides *(B)*, and the number of identified glycoproteins *(C)*. Each dot represents an individual replicate. *D*, Scatter plot showing the distribution of glycopeptide molecular weights in the retentate and filtrate fractions. Horizontal lines indicate the median values for each group.

**Supplemental figure S9. Overview of glycoproteome coverage and quantitative consistency in normal and HCC serum samples.** *A,* Box plots showing the numbers of identified glycopeptides (GPs) per sample for normal (n = 23) and hepatocellular carcinoma (HCC; n = 24) serum samples. *B,* Bar charts summarizing the proportion of MS/MS spectra containing diagnostic oxonium ions for normal and HCC samples, reflecting glycopeptide enrichment efficiency. *C,* Heat-map representation of quantified glycopeptides across all serum samples. Glycopeptide intensities were normalized and hierarchically clustered, with samples grouped according to clinical classification. Color intensity indicates relative glycopeptide abundance. *D,* Functional enrichment analysis of differentially expressed glycopeptides that were significantly up-regulated in HCC, categorized according to Gene Ontology terms, including molecular function, cellular component, and biological process.

**Supplemental figure S10. Representative annotated MS/MS spectra supporting site-specific glycopeptide identification of A2MG.** *A–B,* Annotated spectra of A2MG glycopeptides from the 869 glycosite (Asn-869), including H4N3S1 *(A)* and H6N3S1 *(B)*, identified in HCC samples. *C–D,* Annotated spectra of A2MG glycopeptides from the 1424 glycosite (Asn-1424), including H5N4S2F1 *(C)* and H5N5F1 *(D)*, identified in normal samples. Annotated spectra were generated using the gLabel software.

**Supplemental figure S11. Site-specific glycan profiling of TRFE identified in normal and HCC.** *A,* Heatmap showing all the identified glycans at the glycosite Asn-491, Asn-630, and Asn-432 of TRFE. *B*, Heatmap summarizing site-specific glycan profiles of TRFE, grouped by TRFE protein level, the 491NHC glycosite, and the 432NJS glycosite. Values are displayed as z-scores. *C*, Box plot comparing the TRFE protein level between normal and HCC samples, calculated using intensity values aggregated across all identified glycosites. *D*, Box plot showing the ratio of glycan intensity between the 491NHC and 432NJS glycosites (491/432) for TRFE in normal and HCC samples. Statistical significance was determined by Student’s t-test. *p* < 0.05 (*), *p* < 0.01 (**), *p* < 0.001 (***), and not significant (n.s.).

**Supplemental figure S12. Site-specific glycopeptide changes in up-regulated glycoproteins in HCC serum.** *A,* Heatmap showing site-specific glycosylation patterns of representative up-regulated glycoproteins, including Asn-58 of CO3, Asn-177 of IGHG4, Asn-127 of LUM, Asn-72 and Asn-296 of MSP, and Asn-238, Asn-253, and Asn-352 of SERPING1, across normal and HCC serum samples. Glycopeptide abundances were log₂-transformed and scaled by row to highlight relative changes between conditions. *B,* Box plots of 16 representative intact N-glycopeptides derived from the sites shown in (a), selected based on statistically significant differential abundance (p < 0.05) after excluding glycopeptides requiring extensive imputation. The box plots represent LC–MS–derived quantitative abundances of site-specific glycopeptides from CO3 (n = 5), IGHG4 (n = 6), LUM (n = 2), HGFL (n = 1), and IC1 (n = 2), illustrating consistent up-regulation in HCC serum samples.
